# Classification of Brainstem Gliomas Based on Tumor Microenvironment Status

**DOI:** 10.3390/cancers15174224

**Published:** 2023-08-23

**Authors:** Xiong Xiao, Xiaoou Li, Yi Wang, Changcun Pan, Peng Zhang, Guocan Gu, Tian Li, Zhuang Jiang, Yang Zhang, Liwei Zhang

**Affiliations:** 1 Department of Neurosurgery, Beijing Tiantan Hospital, Capital Medical University, Beijing 100070, China; neurocomputing@163.com (X.X.); leexiaou@163.com (X.L.); wydeqingsu@163.com (Y.W.); pcctt2010@163.com (C.P.); zhang_roc@163.com (P.Z.); gugc2018@163.com (G.G.); lt_ttyy@163.com (T.L.); jzq116@126.com (Z.J.); 2 China National Clinical Research Center for Neurological Diseases, Beijing 100070, China; 3 Beijing Neurosurgical Institute, Beijing Tiantan Hospital, Capital Medical University, Beijing 100070, China

**Keywords:** brainstem glioma, tumor microenvironment, classification, survival

## Abstract

**Simple Summary:**

The tumor microenvironment (TME) is vital in tumor biology, impacting tumor recurrence, prognosis, and treatment response. Brainstem gliomas (BSGs) are challenging gliomas that originate in the brainstem and have distinct clinical and genomic profiles from other brain gliomas. However, the inter-tumor heterogeneity of the TME in BSGs and its correlation with clinical and biological characteristics remain unknown, hindering the development of novel BSG therapies. In this study, we utilized transcriptional data from a BSG cohort and employed established signatures to assess the TME status and classify BSGs accordingly. Subsequently, we found an association between the TME classification and patient prognosis as well as the tumor phenotype. Furthermore, we explored key genes or radiomics features that can determine classification and potentially facilitate clinical applications. This research aims to enhance our understanding of TME heterogeneity in BSGs and to provide insights for improving diagnosis and treatment.

**Abstract:**

The inter-tumor heterogeneity of the tumor microenvironment (TME) and how it correlates with clinical profiles and biological characteristics in brainstem gliomas (BSGs) remain unknown, dampening the development of novel therapeutics against BSGs. The TME status was determined with a list of pan-cancer conserved gene expression signatures using a single-sample gene set enrichment analysis (ssGSEA) and was subsequently clustered via consensus clustering. BSGs exhibited a high inter-tumor TME heterogeneity and were classified into four clusters: “immune-enriched, fibrotic”, “immune-enriched, non-fibrotic”, “fibrotic”, and “depleted”. The “fibrotic” cluster had a higher proportion of diffuse intrinsic pontine gliomas (*p* = 0.041), and “PA-like” tumors were more likely to be “immune-enriched, fibrotic” (*p* = 0.044). The four TME clusters exhibited distinct overall survival (*p* < 0.001) and independently impacted BSG outcomes. A four-gene panel as well as a radiomics approach were constructed to identify the TME clusters and achieved high accuracy for determining the classification. Together, BSGs exhibited high inter-tumor heterogeneity in the TME and were classified into four clusters with distinct clinical outcomes and tumor biological properties. The TME classification was accurately identified using a four-gene panel that can potentially be examined with the immunohistochemical method and a non-invasive radiomics method, facilitating its clinical application.

## 1. Introduction

Brainstem gliomas (BSGs) are a group of gliomas originating in the brainstem [1] that exhibit distinct clinical characteristics and molecular features from gliomas located in the cerebrum [1,2]. In particular, nearly half of the BSGs are diffuse intrinsic pontine gliomas (DIPGs) that represent the deadliest brain cancers in children with very low median survival of less than 12 months [3,4]. Due to the vital functions of the brainstem, surgical resection is quite challenging [5]. Meanwhile, radiotherapy usually provides limited benefits to patients’ survival [6,7]. Additionally, Temozolomide-based chemotherapy has not proven effective in DIPGs, due to their common lack of hyper-methylation in gene O-6-Methylguanine-DNA methyltransferase [8]. The recent success of immunotherapy in hematopoietic malignancies and a variety of solid tumors have stimulated the development of novel immunotherapeutic approaches in treating BSGs, and some have generated encouraging results [9,10,11,12]. Immunotherapy in theory provides a promising treatment option for BSGs due to its ability to precisely target tumor cells while causing minimal disruption to normal neural structures [13]. However, there is no immunotherapeutic approach that has proven clinically effective against BSGs. Therefore, novel therapeutics are urgently needed for this type of brain tumor.

A comprehensive understanding of the tumor microenvironment (TME) in BSGs will facilitate the development of novel therapeutic approaches against BSGs, since TME plays a pivotal role in tumorigenesis and tumor progression [14] and engages key molecular pathways determining the response to therapeutics such as radiation and immunotherapies [15,16,17]. Several studies have revealed DIPG as an immune-cold tumor, exhibiting reduced infiltration of immune cells and a lower expression of immunosuppressive molecules relative to glioblastoma (GBM) [13]. These findings are consistent with previous reports that DIPG is innately resistant to an immune checkpoint blockade [12] and also suggest their unique TME features from gliomas in the other part of the brain. Meanwhile, recent advances in the TME investigation have promoted the development of a series of TME classification models for a variety of malignancies [15,18,19,20]. Based on inter-tumoral variations in TME components, these models provided clinicians with a useful tool for predicting therapeutic repones and patients’ survival, as well as facilitating the development of novel therapeutics [15,18,19,20]. In gliomas, a three-subtype TME model suggests that its IS3 subtype would have a better response to mRNA-based anti-tumor vaccination [19]. However, as a unique group of gliomas, the TME classification for BSGs has not been reported.

BSGs are also a heterogeneous group of neoplastic disorders, as evidenced by reports detailing variations in patient demographics, clinical features, imaging characteristics, and molecular biology [1,2,21,22,23]. It is widely known that BSGs are divided into DIPGs and non-DIPGs, or H3K27M-mutant and H3K27-wildtype, according to their imaging and genomics findings [2]. Choux et al. defined a four-subtype classification based on tumors’ locations and growth patterns, which proved useful in surgical case selections [21]. A five-subtype multimodality imaging-based radiological classification can help in selecting optimal treatment strategies for pediatric DIPGs [24]. We previously grouped BSGs into four subtypes according to their methylation status, and each type represented distinct clinical profiles and genomic landscapes [2]. We uncovered gene sets that were enriched in the methylation cluster H3-Medulla, involving immune-related pathways such as interferon-gamma/alpha signaling, the IL-10 pathway, and the CTLA-4 pathway [2]. However, the direct correlation of TME with clinical profiles and biological characteristics of BSGs was not examined in the study [2] or reported previously. Moreover, whether BSGs also exhibit inter-tumor heterogeneity in TME and how significant that heterogeneity is remain elusive.

Recently, Bagaev et al. utilized a list of gene expression signatures (GESs) that encompass four key features of TME to classify cancer: anti-tumor immune response, pro-tumor immune response, angiogenesis/fibrosis, and malignant cell properties [15]. The authors identified a pattern of TME subtypes, which persist across various types of cancers and are associated with immunotherapy response. However, the investigation did not include gliomas. Herein, we exploited these GESs to classify BSGs and revealed rather high inter-tumor heterogeneity in the TME among BSGs. As such, BSGs are grouped into four TME clusters with distinct clinical outcomes and differential tumor biological features. Furthermore, the TME classification system has independent prognostic value and can be identified using a four-gene panel, potentially with the routine immunohistochemical (IHC) method or by a non-invasive radiomics approach.

## 2. Materials and Methods

### 2.1. Patient Inclusion and RNA Sequencing

A total of 98 BSG cases were included in this study (Figure S1). Among them, 75 patients had RNA sequencing (RNA-seq) profiles that were published in our previous study [2], and RNA-seq profiles of 23 cases were firstly included in this study. The follow-up information of all these patients was updated as of December 2022. The demographic variables and mutation information, as well as other clinical variables, were extracted from the previously published literature [2]. The study was approved by the Institute Review Board (IRB) of Beijing Tiantan Hospital. Informed consent was waived by the IRB because of the retrospective nature and previously obtained consent in the previous study.

RNA-seq was performed on the Illumina HiSeq X Ten platform by Genetron Health (Beijing, China) [2]. Alignment was carried out using either STAR or hisat2, while gene expression profiling was performed using Cufflinks. The presence of batch effects between the expression profiles of the two RNA sequencing batches was evaluated using the Umap method (R package umap v0.2.7.0). If significant batch effects were observed, Combat_seq was employed to mitigate them (Figure S2) [25]. Following this step, the raw count data were transformed into transcripts per million (TPM) values for further analysis.

### 2.2. TME Status Estimation and Clustering

A list of GESs that encompass four key features of TME: anti-tumor immune response, pro-tumor immune response, angiogenesis/fibrosis, and malignant cell properties [15] were utilized to estimate the TME status of BSGs with the method of single-sample gene set enrichment analysis (ssGSEA). The results of ssGSEA were subjected to consensus clustering using the R package ConsensusClusterPlus (v1.51.1). The clustering was conducted by employing partitioning around the medoid and determining the distances between samples based on the Euclidean distance method. The optimal number of clusters was identified by analyzing the consensus cumulative distribution function (CDF) plot and its delta area plot (Figure S3). A heatmap was generated using the R package Pheatmap (v1.0.12) to depict the distribution of the ssGSEA results of the GESs among the clusters.

### 2.3. Estimation of Tumor Biological Features and Radiosensitivity

R package PROGENy (version 1.20.0) was used to evaluate the activity of 14 pathways commonly implicated in tumors based on RNA-seq profiles [26]. Radiosensitivity was estimated using the radiosensitivity index and radiotherapy risk signature [27,28], both inversely correlated with survival time after radiotherapy.

### 2.4. Identification of Key Genes Determining TME Clusters

Given that the TME classification is formed by two orthogonal properties: immune (immune-enriched vs. immune-depleted) and fibrosis (fibrotic vs. non-fibrotic) [15], we first examined the differential gene expression between the two immune-enriched clusters (“immune-enriched, fibrotic” and “immune-enriched, non-fibrotic”) and the two immune-depleted clusters (“fibrotic” and “depleted”) through the R package limma (v3.15), and genes with FDR < 0.05 and |Log2FoldChange| > 3 were chosen for further analyses. Subsequently, we performed the binary least absolute shrinkage and selection operator (LASSO) regression analysis on these genes, and those with non-zero coefficients were selected as key genes. An “immune-enriched” score was subsequently generated based on the expression levels and coefficients of key genes, using the formula score = ∑ (βi × Expi), where βi represents the corresponding regression coefficient of a gene, and Expi represents the TPM value of the gene. With the same approach, the key genes and according “fibrotic” score were established, respectively.

The established “immune-enriched” and “fibrotic” scores were further validated in BSG cases in five external datasets, namely the Pacific Pediatric Neuro-Oncology Consortium (PNOC) dataset, the Children’s Brain Tumor Tissue Consortium (CBTTC) dataset, the Pediatric Cancer Genome Project (PCGP), the Real-time Clinical Genomics (RTCG) dataset, and our other unpublished RNA-seq dataset comprising 19 BSGs (Figure S1). All the included cases were first stratified as the high- and low-expression groups based on the scores from ssGSEA on GESs related to the immune-enriched or fibrosis properties. Receiver operating characteristic (ROC) curves were plotted, and the area under the curve (AUC) was calculated to demonstrate the accuracies of the “immune-enriched” and “fibrotic” scores in discriminating between the according high- and low-expression groups.

### 2.5. Multiplex Immunofluorescence Staining

In order to validate whether the key genes have differential expressions between the corresponding clusters, a total of 31 cases with available formalin-fixed paraffin-embedded samples were subjected to multiplex immunofluorescence staining. An OPALTM 7-color manual immunofluorescence staining kit (NEL811001KT, Akoya Bioscience, Marlborough, MA, USA) was used to perform multiplex immunofluorescence staining detection on paraffin-embedded tissues. After deparaffinization and hydration, an optimized program for each antigen was applied to the slides (Tables S1 and S2). Slides were scanned on the Vectra system (Vectra Polaris 1.0.7, Akoya Bioscience, Marlborough, MA, USA) and analyzed using the Inform software (2.4.6, Akoya Bioscience, Marlborough, MA, USA). The positive rate of each marker was defined as the number of positive cells’ nuclei divided by the total number of nuclei in the section or the positive area divided by the total tissue area if the antigen was distributed in intercellular space. The counting of positive cells and the calculation of the staining area were performed by two neuropathologists, and the results were subsequently cross-validated.

### 2.6. Generation of Radiomics Models for Identifying TME Clusters

A total of 88 cases with available magnetic resonance imaging (MRI) data were included in the analysis. The T1-weighted, T2-weighted, and T1-weighted contrast sequences were previously published, and they were retrieved for this study [29]. The delineation of the tumor contour on each sequence was carried out by an experienced neurosurgeon using Slicer (version 5.2.1) and subsequently confirmed by another neurosurgeon. The extraction of radiomics features from the original images and images subjected to wavelet filtering and Laplacian of Gaussian filter was performed using Pyradiomics (version 3.0.1) [30]. Thus, a total of 3390 radiomics features were extracted.

Two predictive models were developed for the two TME properties: immune-enriched and fibrotic. The included cases were randomly divided into a training set and a test set using a 6:4 ratio. Within the training set, the radiomics features were compared between cases with high and low expression in the according TME property using Wilcoxon rank sum tests. Only features with a *p*-value < 0.05 were retained. Secondly, retained features were further selected using a random forest algorithm, resulting in no more than 10 features. Thirdly, the support vector machines (SVM) algorithm with the eps-regression type and a radial kernel based on these selected features were used to develop prediction models for the TME properties. The R package e1071 (version 1.7) was used for the analysis. ROCs were plotted, and AUCs were calculated to assess the model accuracy for the discrimination.

### 2.7. Statistical Analysis

Statistical analyses were performed using R (version 4.1.2). For continuous variables following a normal distribution, means and standard deviations were used for description, and t tests or ANOVAs were used for intergroup comparison. For continuous variables following a non-normal distribution, quartiles were used for description, and the Mann-Whitney U test or Kruskal-Wallis test was used for intergroup comparisons. Frequencies and percentages were calculated for categorical variables, and chi-square tests were used for intergroup comparisons. For pairwise comparisons between subgroups, *p* values were corrected via the Benjamini–Hochberg method. Overall survival was estimated and compared using the Kaplan-Meier method, and risk factors for prognosis were determined with univariate and multivariate Cox regression (R package survminer v0.4.9).

## 3. Results

### 3.1. BSGs Are Grouped into Four Clusters Based on TME Status

**Figure 1:**
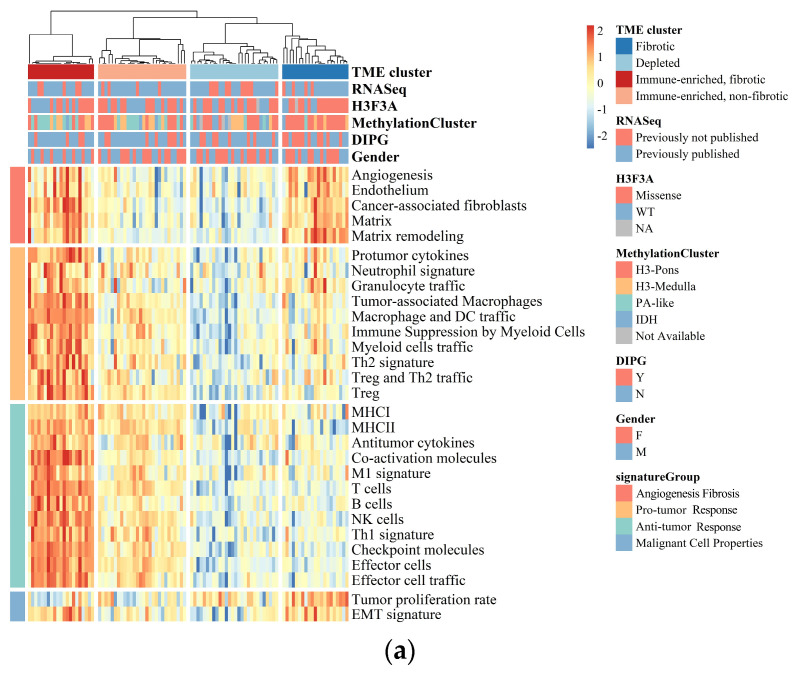

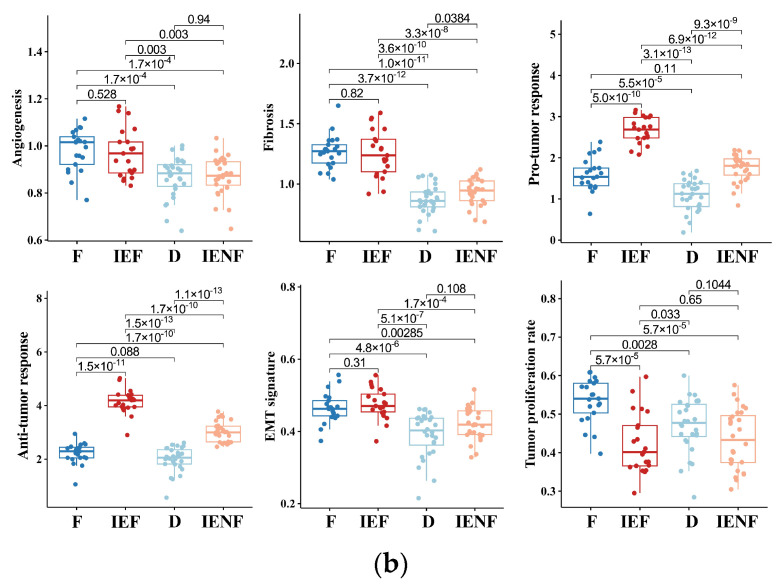
BSGs are grouped into four clusters based on their TME status. (**a**) A heatmap for results of consensus clustering based on the TME-related GESs among 98 BSG cases; (**b**) comparison of activities between clusters in key components of the pan-cancer TME GESs; the activities were measured as scores of the according GESs. F: fibrotic; IEF: immune-enriched, fibrotic; D: depleted; IENF: immune-enriched, non-fibrotic.

BSGs are a group of heterogeneous malignancies located in the brainstem, with distinct genetic mutations from gliomas in the other part of the brain [1,2]. Herein, we aimed to understand their heterogeneity in TME through the classification of BSGs utilizing a list of pan-cancer-conserved GESs that encompass four key features of TME: anti-tumor immune response, pro-tumor immune response, angiogenesis/fibrosis, and malignant cell properties [15]. A total of 98 BSG cases were included for the classification (Figure S1), resulting in four TME clusters: “immune-enriched, fibrotic (IEF)”, “immune-enriched, non-fibrotic (IENF)”, “fibrotic (F)”, and “depleted (D)” (Figure 1a and Figure S3).

The “fibrotic” cluster was characterized by the highest expression of genes related to angiogenesis/fibrosis, whereas it exhibited relatively low activity in the immune response (Figure 1b). The “immune-enriched, fibrotic” cluster showed increased activity in the angiogenesis/fibrosis and immune response, while the “depleted” cluster displayed the lowest levels of these TME properties (Figure 1b). Additionally, the “immune-enriched, non-fibrotic” cluster displayed relatively increased activity in the immune response, whereas it exhibited a lower level of angiogenesis/fibrosis than the “immune-enriched, fibrotic” and “fibrotic” clusters (Figure 1b). In terms of malignant cell characteristics, the “fibrotic” BSGs exhibited the highest tumor proliferation rate, while the “fibrotic” and “immune-enriched, fibrotic” clusters showed enhanced epithelial–mesenchymal transition (EMT) activity compared to the other clusters (Figure 1b). These results are consistent with the previous findings on the other malignancies [15], suggesting the TME classification is also conserved in BSGs.

### 3.2. The TME Clusters Are Correlative with BSG Clinical and Molecular Features

**Figure 2:**
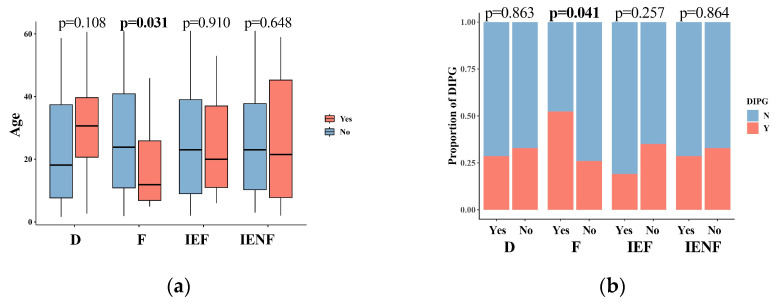

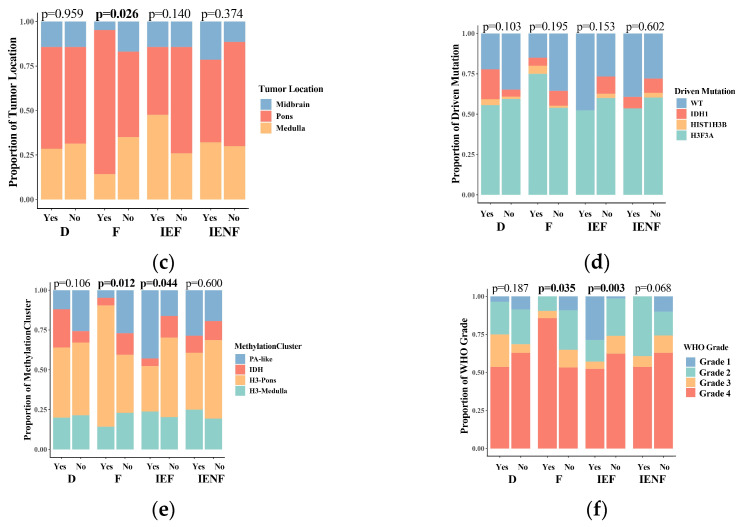
The TME clusters are correlated with the clinical and molecular features of BSGs. Comparisons between clusters in patients’ ages (**a**); in proportions of DIPG (**b**), tumor locations (**c**), driver mutations (**d**), methylation clusters (**e**), and WHO grades (**f**). F: fibrotic; IEF: immune-enriched, fibrotic; D: depleted; IENF: immune-enriched, non-fibrotic; bold font: *p* < 0.05.

We next correlated the clinical characteristics and molecular features of BSGs with the TME clusters (Figure 2). We observed that diffuse intrinsic pontine gliomas (DIPGs) accounted for nearly half of the fibrotic cluster; the proportion was significantly higher than that observed in the other clusters (Figure 2b, *p* = 0.041). Since a younger age, tumors located in the pons, the methylation cluster of H3-Pons, and WHO grade 4 tumors are highly correlated with the DIPG type [2,3], and they were also enriched in this cluster (Figure 2a,c,e,f). On the other hand, the fibrotic cluster constituted 35.5% of all DIPG tumors and represented the most common TME type in DIPG (Figure S4A). We also observed that the PA-like tumors were enriched in the “immune-enriched, fibrotic” cluster (Figure 2, *p* = 0.044), leading to a higher proportion of benign tumors (WHO grade 1) in this cluster (Figure 2, *p* = 0.003), since the PA-like tumors mainly comprise piloctyic astrocytoma and ganglioglioma [2]. Additionally, 42.9% of PA-like tumors exhibited the “immune-enriched, fibrotic” property (Figure S4B). Together, we observed the enrichment of DIPGs in the “fibrotic” type and the PA-like tumors in the “immune-enriched, fibrotic” cluster, thereby revealing the TME property for these two BSG types.

### 3.3. The TME Clusters Predict Outcomes in BSG Patients

**Figure 3:**
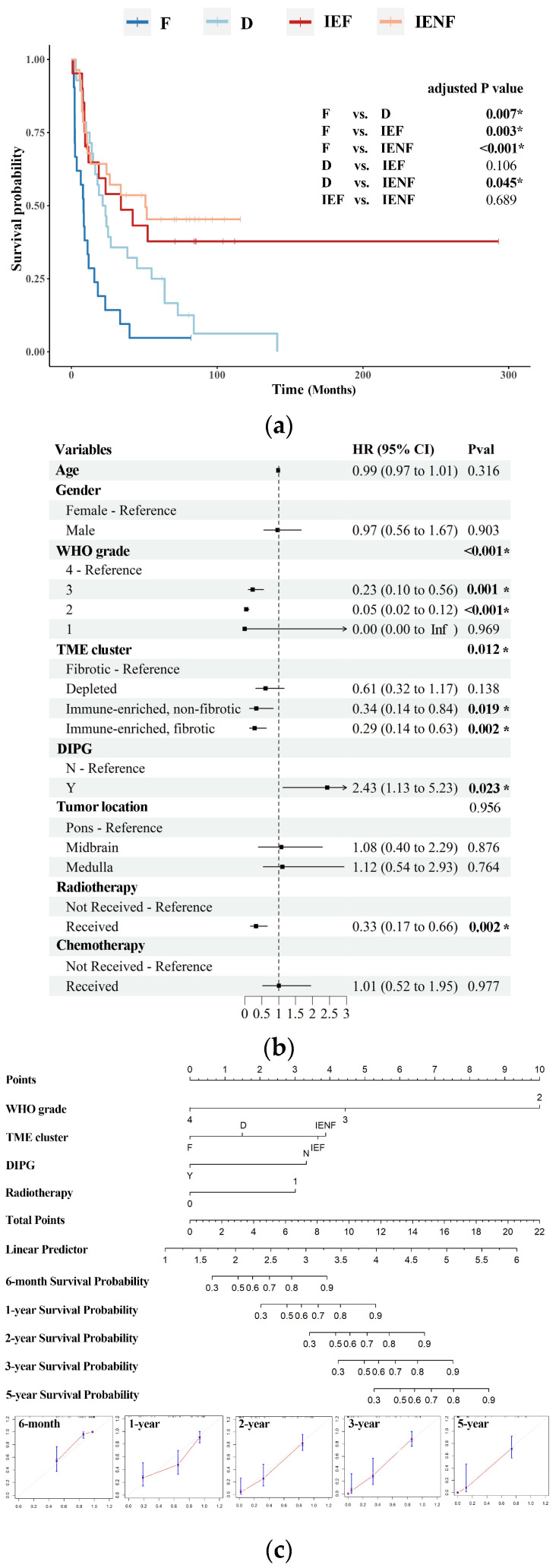
The TME clusters predict outcomes in BSG patients. (**a**) The Kaplan–Meier analysis showing the survival differences among the TME clusters; (**b**) the multivariate Cox regression showing variables including DIPG diagnosis, the TME clusters, received radiotherapy, and WHO grades as independent risk factors for prognosis; (**c**) a nomogram based on WHO grades, DIPG diagnosis, received radiotherapy, and the TME clusters. F: fibrotic; IEF: immune-enriched, fibrotic; D: depleted; IENF: immune-enriched, non-fibrotic; * *p* < 0.05.

We next investigated the prognostic value of the TME classification system. With a medial follow-up time of 18.8 months, the four TME clusters exhibited distinct overall survival (*p* < 0.001, Figure 3a). Specifically, the “fibrotic” cluster displayed the poorest outcome with a median survival of 8.3 months, whereas the “depleted” cluster showed a better prognosis relative to the “fibrotic” cluster, with a median survival of 21.4 months. Both of the two immune-enriched clusters exhibited more favorable outcomes; the median survival is 34.0 and 50.8 months, respectively, in the “immune-enriched, fibrotic” and “immune-enriched, non-fibrotic” clusters.

The TME clusters, driver mutations, WHO grades, tumor locations, and DIPG diagnoses had an impact on patients’ survival, as indicated by univariate Cox regression (Table S3). These variables were included in the multivariate Cox regression analysis, except for driver mutations, which have already been incorporated with WHO grades [31]. Demographic variables and “whether received radiotherapy/chemotherapy or not” were also included as they were potential factors for patients’ survival [6,32]. As shown in Figure 3b, the TME classification emerged as an independent factor associated with overall survival, along with the WHO grade, DIPG, received radiotherapy, and common prognosticators for BSGs [1,6,31,32]. Furthermore, a nomogram was constructed by integrating these variables, and it showed relatively satisfactory accuracy in predicting the outcomes of malignant BSGs (Figure 3c).

### 3.4. The TME Clusters Exhibit Distinct Molecular Pathway Activity and Radiation Sensitivity

**Figure 4:**
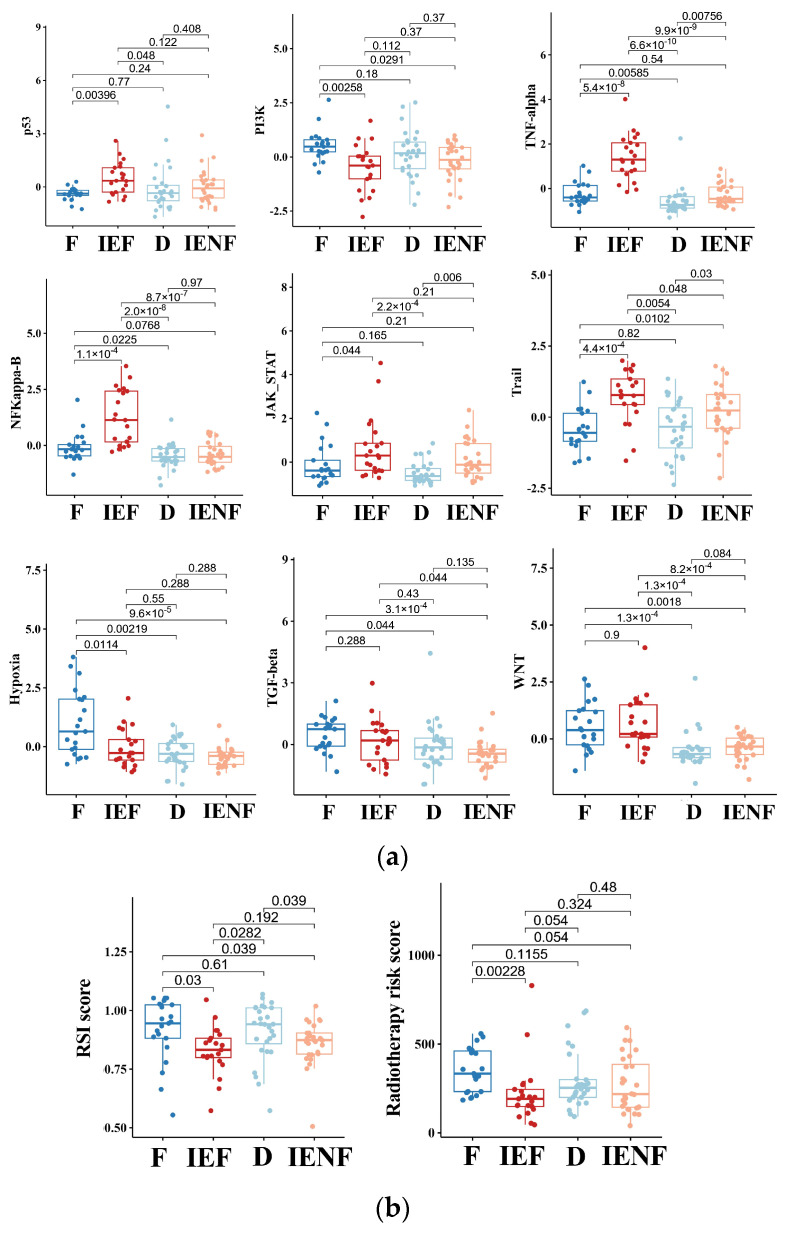
The TME clusters exhibit distinct molecular pathway activity and radiation sensitivity. (**a**) Comparisons of activities between clusters in the PROGENy pathways that are commonly implicated in tumors; (**b**) comparisons of radiosensitivity between clusters and the lower scores correlating with better outcomes following radiotherapy. F: fibrotic; IEF: immune-enriched, fibrotic; D: depleted; IENF: immune-enriched, non-fibrotic.

We next explored molecular mechanisms underlying the distinct outcomes exhibited by the four clusters. As suggested previously (Figure 1), the “fibrotic” cluster exhibited the highest level of angiogenesis, tumor cell proliferation, and EMT activity, which correlate with its worst-outcome phenotype. Meanwhile, this cluster also exhibited relatively high activity in the molecular pathways related to PI3K, hypoxia, TGF-β, and WNT (Figure 4a). Abnormal activations of these pathways are typical hallmarks of glioma [33,34,35,36], contributing to glioma progression and malignant phenotypes, such as proliferation, stem cell property, invasion, and angiogenesis [33,35,36,37]. In particular, the pathways of TGF-β and WNT are highly correlated with the process of fibrosis [33,35,37], and both pathways’ activities are elevated in the two fibrotic clusters, “fibrotic” and “immune-enriched, fibrotic”, suggesting that these pathways would be intrinsic mechanisms for the fibrotic phenotype in glioma, thus requiring further exploration.

In contrast, we observed that the two immune-enriched clusters, “immune-enriched, fibrotic (IEF)” and “immune-enriched, non-fibrotic (IENF)”, showed relatively high activities in the pathways related to the anti-tumor immune response (TNF-α), tumor inflammation (NF-κB and JAK-STATs), and apoptosis (Trail), as compared with the other clusters, thus providing molecular clues for their better outcomes (Figure 4a).

Since radiotherapy is a standard treatment for most BSGs [1], we next asked whether these clusters exhibited a difference in sensitivity to radiation, thus leading to their distinct outcomes. Therefore, we utilized two independent radiation-resistant signatures, both of which have shown high accuracy in predicting radiotherapeutic outcomes in glioma [27,28]. As a result, we observed that the “fibrotic” cluster showed a relatively high resistance score as compared with the two immune-enriched clusters (Figure 4b), reflecting that the “fibrotic” cluster is more resistant to radiotherapy. With respect to mechanisms underlying radiation resistance, the TP53 pathway is the core mechanism involved in radiation response, and its activity was consistently decreased in the cluster (Figure 4a). Furthermore, other pathways such as hypoxia, WNT, TGF-β, and PI3K are reportedly linked to glioma radio-resistance by modulating the TP53 pathway [33,38,39,40], and their activities were also elevated in the cluster (Figure 4a), indicating an intertwined effect of these pathways on the BSG radiotherapy response.

### 3.5. A Four-Gene Panel Determines the TME Classification

For the better clinical application of the TME classification system, we tried to identify a few key genes that can distinguish among the clusters and be examined using routine clinical assays as well. Since the classification is formed by two orthogonal axes, the immune (immune-enriched vs. immune-depleted) and fibrosis (fibrotic vs. non-fibrotic) [15], we first identified the differential gene expression related to the immune and fibrosis axes, respectively (Figure S6).

We next utilized the method of LASSO regression on filtered genes for dimensionality reduction and determining the key genes associated with each axis (Figure S6). As a result, we revealed that CD3E was a key gene overexpressed in the “immune-enriched” clusters, whereas COL3A1, CA9, and MMP1 were relatively highly expressed in the “fibrotic” clusters. Based on the coefficients computed from the LASSO regression, two signatures were constructed: fibrotic score = COL3A1 × 0.0010181389 + MMP1 × 0.0257429451 + CA9 × 0.0005309276; immune-enriched score = CD3E × 0.01988081.

**Figure 5:**
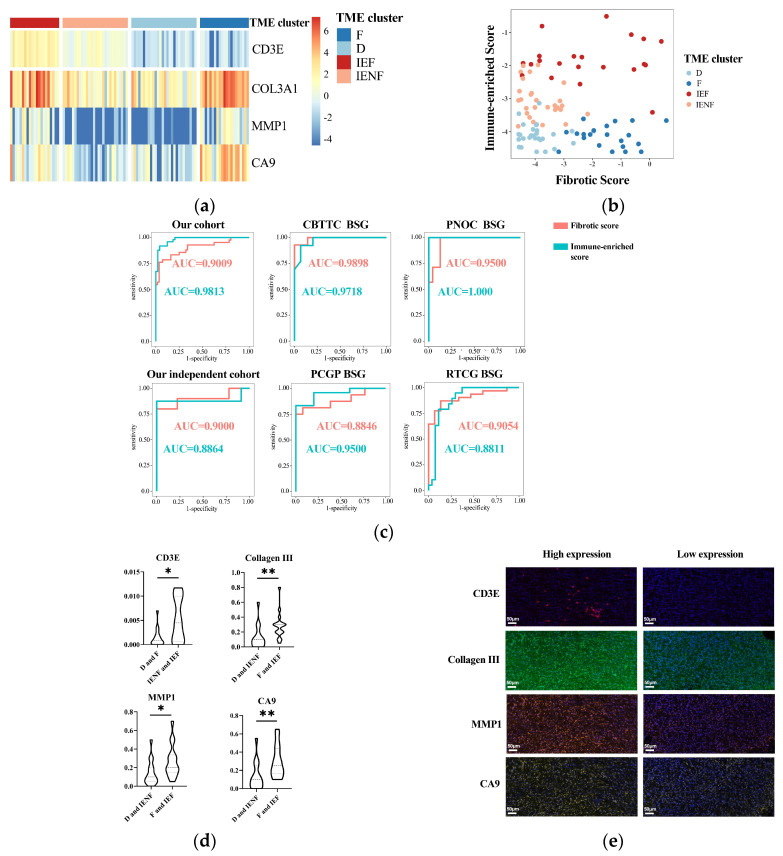
A four-gene panel determines the TME classification. (**a**) A heatmap showing distinct expression of the four genes among the TME clusters; (**b**) the immune-enriched or fibrotic scores based on the four-genes’ expressions showing good discrimination ability for the TME clusters; (**c**) ROC curves showing high accuracy of the immune-enriched or fibrotic scores for identifying the according clusters in six independent cohorts; (**d**) differences of four genes’ expressions between the TME clusters observed using multidimensional fluorescence staining; (**e**) representative multidimensional fluorescence staining images of four genes’ expressions: CD3E in red, Collagen III in green, MMP1 in tangerine, and CA9 in gold. * *p* < 0.05; ** *p* < 0.01; F: fibrotic; IEF: immune-enriched, fibrotic; D: depleted; IENF: immune-enriched, non-fibrotic.

The expression of the four-gene panel can classify the BSG into four TME clusters (Figure 5a,b). The panel exhibited rather high accuracy for classification with the value of AUC (area under the receiver operating characteristic curve) as 0.9813 and 0.9009 for the immune and fibrotic axes, respectively. This performance was also validated in another five external BSG datasets (Figure 5c and Figure S5), further indicating the high accuracy of the panel for TME classification.

Finally, we exploited the IHC method to assay the expression of the four key genes in tumor tissues for validating and examining the possibility of using routine clinical assays for the determination of TME classification. As a result, the expression of the four genes was significantly elevated in the according clusters (Figure 5d,e), suggesting the reliability of these key genes and their potential as IHC markers for TME classification.

### 3.6. Radiomics Features Related to TME Clusters

**Figure 6:**
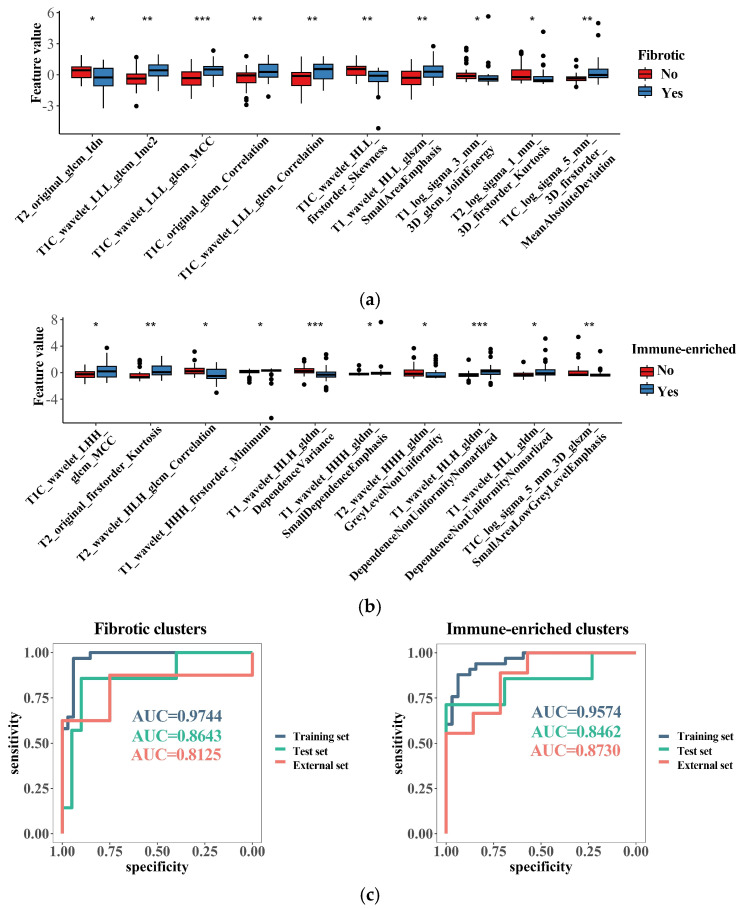
Radiomics features related to the TME clusters. (**a**) The top 10 most important radiomics features selected with random forest algorithm for identifying the “fibrotic” clusters; (**b**) the top 10 most important radiomics features selected with random forest algorithm for identifying the “immune-enriched” clusters; (**c**) discrimination ability of generated radiomics models for identifying the “fibrotic” or “immune-enriched” clusters. * *p* < 0.05; ** *p* < 0.01; *** *p* < 0.001.

After the selection via the methods of the Mann–Whitney U test and random forest selection, a list of 10 radiomic features emerged as the most important indicators for identifying the immune-enriched or fibrotic clusters (Figure 6a,b). Interestingly, most of the fibrosis-related features correlated with increased texture complexity, and the majority of immune-related features reflected high variation in the gray level, implying underlying links between the TME clusters and MRI features. SVM models integrating these features were successfully built for identifying the “immune-enriched” and “fibrotic” clusters, respectively. The two models demonstrated a rather high accuracy in identification according to clusters in the training, test, and external datasets (Figure 6c). Therefore, the TME clusters can be identified with non-invasive MRI analysis, which would facilitate the wide clinical application of TME classification.

## 4. Discussion

Mounting evidence has supported the fundamental role of TME in tumor genesis and progression [15,16], thus significantly impacting the tumor recurrence, prognosis, and therapeutic response [17]. Additionally, TME classification models have been reported for a variety of tumor types, which have been reported as valuable for predicting prognosis and selecting treatment [15,19,20]. Therefore, a comprehensive understanding of TME is essential for the precise diagnosis and thus better management of BSGs. BSGs are reported to be a group of tumors with significant heterogeneity [1,2,21], and we have found differences in tumor immune microenvironments between cases in previous studies [2]. However, there is still a lack of systematic research on the heterogeneity of BSGs in terms of TME, and TME classification for BSGs has not been systematically reported before this study.

Herein, we utilized a list of pan-cancer-conserved and TME-related GESs [15] to classify BSGs according to the TME status. Additionally, consistent with the findings on the other malignancies [15], BSGs are also grouped into four clusters, reflecting that the TME classification is conserved in BSGs. Interestingly, we observed the high inter-tumor heterogeneity of TME in BSGs, even in DIPGs, which are often unanimously recognized as immune-cold tumors [13]. These results suggest that the TME classification would be an important indicator for BSG subtyping. Also, we observed that the TME classification was associated with the tumor phenotype, had an independent influence on prognosis, and was correlated with the activities in malignancy-related pathways and radiation sensitivity. Further, a four-gene panel and a radiomics approach were developed to identify the TME clusters. This may be the first study to report a TME classification specifically for BSGs, which may be useful in deepening the understanding of BSGs and improving their precise diagnosis and treatments.

DIPGs comprise a group of BSGs that diffusedly infiltrate pons and commonly harbor a K27M mutation in gene H3F3A [3,4]. It represents one of the deadliest pediatric brain malignancies, with a short medial survival of less than 12 months after diagnosis [3]. It is also universally concerned as an immune-cold tumor [4,13] that is innately resistant to immunotherapy such as an immune checkpoint blockade [2,3,4,41]. In this study, we also observed a higher proportion of the “fibrotic” cluster in DIPGs than the other BSGs (Figure 2b), and on the other hand, the immune-depleted clusters (the “fibrotic” cluster and the “depleted” cluster) constituted the majority of DIPGs (Figure S4A). However, we uncovered nearly 40% of DIPGs grouped into the immune-enriched clusters, suggesting a TME heterogeneity in DIPGs that would be transferred into their distinct clinical outcomes (Figure 3), differential response to radiation sensitivity (Figure 4), and immunotherapies as well. Therefore, the TME classification would impact outcomes in DIPG patients receiving experimental therapeutics and should thus be considered in the design for the according clinical trials. Additionally, the intrinsic mechanisms driving heterogeneity also pose an intriguing question that warrants further exploration.

Several studies have shown TME features as another dimension of prediction systems for prognosis, improving the predictive accuracy of current TNM staging systems [15,42,43]. We also revealed the TME classification as an independent predictor for BSG patients’ outcomes (Figure 3a,b). Accompanying the WHO grading system, DIPG, and radiotherapy, the TME classification provided additional valuable information, allowing for a more precise prediction for prognosis (Figure 3c) and highlighting its clinical significance as a new prognosis prediction system for BSG patients. Meanwhile, since the TME clusters have shown their independent prognostic value, it is suggested that novel treatments that can modify the TME status may have the potential to benefit BSG patients [16,44].

The determination of the GESs requires measuring hundreds of genes, which may restrict the clinical use of the TME classification. As a result, two simplified methods were developed to identify the TME subtypes. We developed a four-gene panel that demonstrated high accuracy in identifying TME clusters in both our cohort and external datasets. The four genes included in the panel were mechanistically reasonable, as CD3E is present in the majority of T cells, while COL3A1 and MMP1 are representative components and enzymes in the fibrotic extracellular matrix environment [15,45,46]. Moreover, since these genes have been validated through IHC to show significant differences between subtypes, it is suggested that the panel is not only reliable but also has the potential to be clinically determined using a common IHC method. Furthermore, an identification approach was developed using radiomics features and the SVM algorithm, which demonstrated satisfactory efficacy in identifying the TME clusters. This finding is consistent with similar research conducted on other types of tumors that show that the TME status can be predicted using radiomics approaches [47]. And future studies will include additional imaging features and more advanced algorithms in order to develop TME prediction models that are more effective and easier to interpret. These models will also be validated in larger sample sizes and across multiple centers.

However, there are some limitations in this study. Firstly, this is a post hoc study utilizing old data, most of which have been published [2], and would contain confounding factors restricting the extension of our result. For instance, the majority of DIPG tumor samples were collected from patients who received debulking surgery at our center [2,23]. Additionally, the DIPGs eligible for debulking surgery contained a higher proportion of MRI-contrast-enhanced appearances than the biopsy-eligible DIPGs that usually appear as homogenously non-contrast images. Therefore, the observation of high TME heterogeneity could be partly attributed to the DIPG type with contrasted MRI appearances. Secondly, due to the rarity of BSG and the difficulty in obtaining tumor tissues using neurosurgical approaches, the sample size in this study is relatively small, which might also introduce biases in the conclusion. A larger multi-center cohort established through international collaboration is thus warranted [6] to better understand the significance of this TME classification. Thirdly, due to the limited number and small scale of BSG trials, a study on the value of TME classification in therapeutic responses is lacking.

## 5. Conclusions

BSGs exhibited a high inter-tumor TME heterogeneity, such that they were classified into four TME clusters with distinct clinical outcomes and tumor biological properties. The TME classification can be well identified using a four-gene panel and a non-invasive radiomics method, thus facilitating its clinical application.

## Data Availability

The data presented in this study are available on request from the corresponding author.

## Supplementary Materials

The following supporting information can be downloaded at https://www.mdpi.com/article/10.3390/cancers15174224/s1, Figure S1: workflow of this study; Figure S2: batch effects were removed using Combat_seq; Figure S3: determination of cluster number; Figure S4: proportions of TME clusters in DIPGs and PA-like tumors; Figure S5: results of consensus cluster of external datasets; Figure S6: procedures of determining key genes; Table S1: antibodies or DAPI and the conditions used for multidimensional fluorescence staining; Table S2: procedures for multidimensional fluorescence staining; Table S3: results of univariate Cox regression.
