# Supplementary material for: Classification of Brainstem Gliomas Based on Tumor Microenvironment Status
Source: Cancers (Basel). 2023 Aug 23;15(17):4224. doi: 10.3390/cancers15174224 (PMC10487167; doi:10.3390/cancers15174224)
Supplement: Supplementary file 1 [file cancers-15-04224-s001.zip › Supl Figures.pdf]

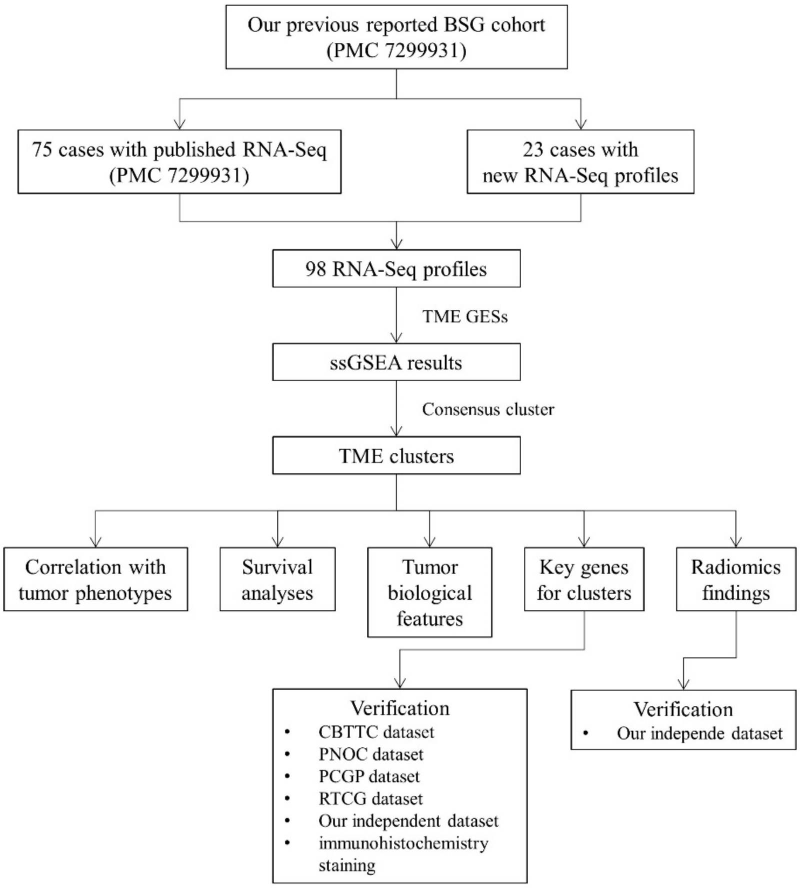

Figure S1. Workflow of this study

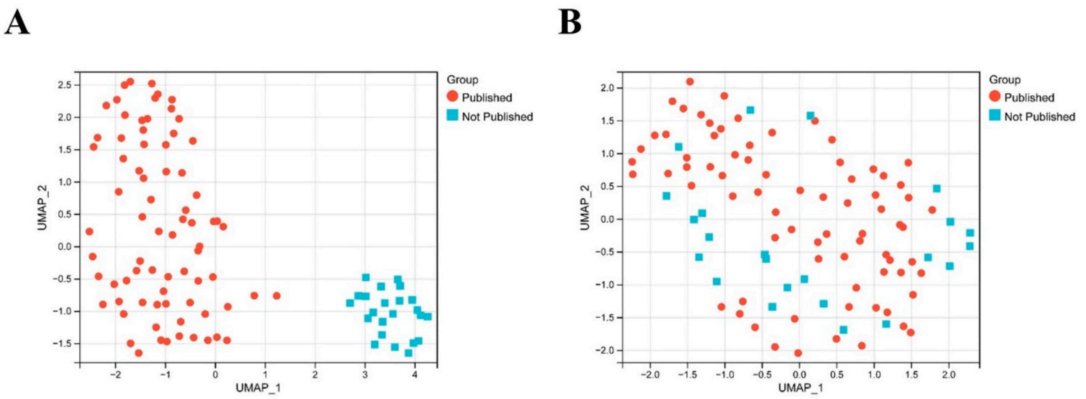

Figure S2. Batch effects were removed using Combat_seq. A: Batch effects observed. B: Batch effects were removed using Combat_seq.

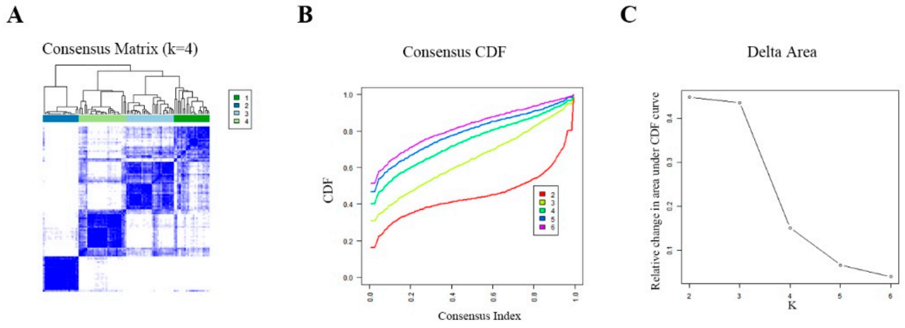

Figure S3. Determination of cluster number. A. Consensus Matrix during clustering. B. Consensus CDF curves. C. Delta area along number of clusters. CDF, cumulative distribution function.

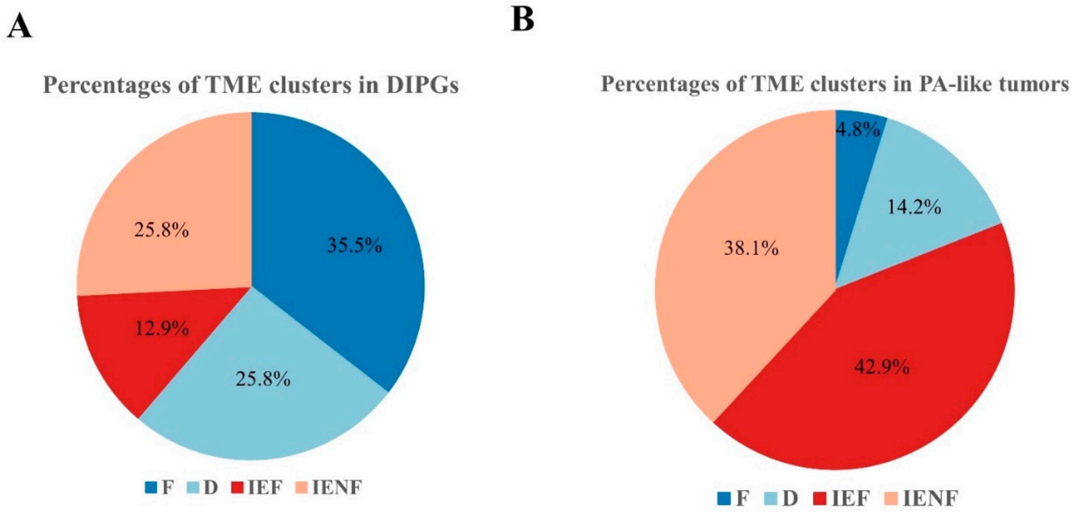

Figure S4. Proportions of TME clusters in DIPGs and PA-like tumors. F: fibrotic; IEF: immune-enriched, fibrotic; D: Depleted; IENF: immune-enriched, non-fibrotic.

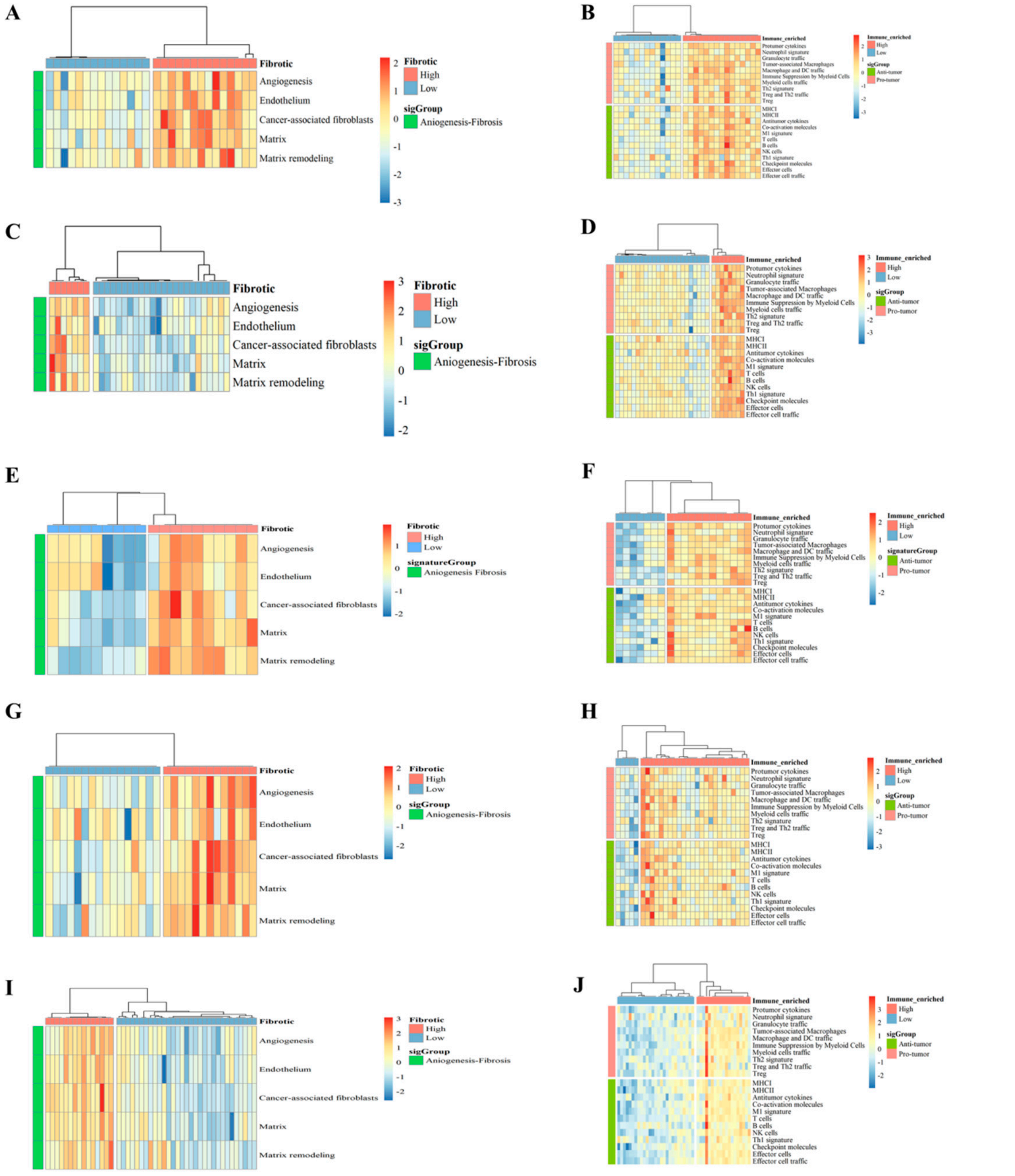

Figure S5. Results of consensus cluster of external datasets. A-B. CBTTC dataset, C-D. Results of PNOC dataset. E-F. Results of our independent cohort. G-H. Results of Pediatric Cancer Genome Project dataset. I-J. Results of Real-time Clinical Genomics dataset.

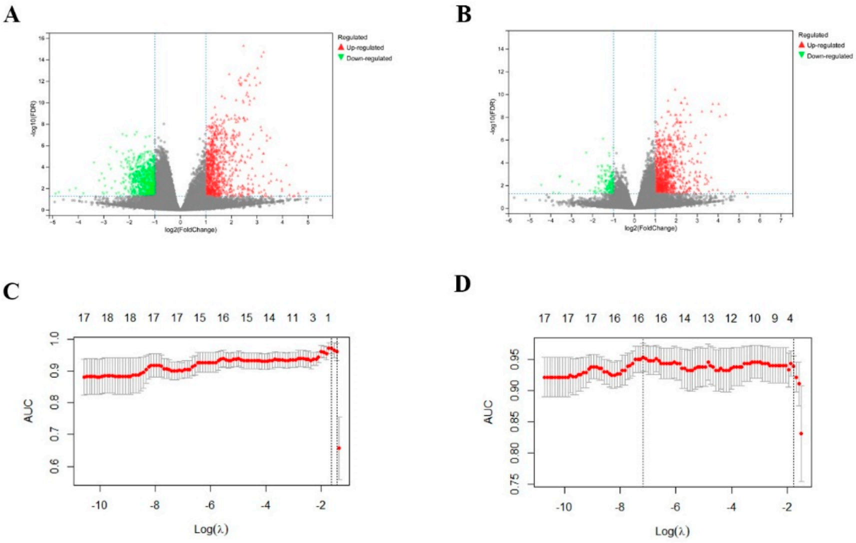

Figure S6. Procedures of determining key genes. A-B. Vocano plots displaying differential gene expression between groups. C-D. LASSO regression procedures (A, C: “fibrotic” and “immune-enriched, fibrotic” clusters vs “depleted” and “immune-enriched, non-fibrotic” clusters; B, D: “immune-enriched, non-fibrotic” and “immune-enriched, fibrotic” clusters vs “depleted” and “fibrotic” clusters)
