# Supplementary material for: Classification of Brainstem Gliomas Based on Tumor Microenvironment Status
Source: Cancers (Basel). 2023 Aug 23;15(17):4224. doi: 10.3390/cancers15174224 (PMC10487167; doi:10.3390/cancers15174224)
Supplement: Supplementary file 1 [file cancers-15-04224-s001.zip › supl tables.pdf]

Table S1. Antibodies or DAPI and the conditions used for multidimensional fluorescence staining

Stain Order	Target	Antibody Clone (host species) or lot# /Company	Dilution	Opal dye (Dilution)	CITY/Nation
**1**	Collagen III	ab23445 (Mouse)/Abcam	1/200	520 (1:100)	Cambridge/UK
**2**	CA9	ab107257 (Mouse)/Abcam	1/400	570 (1:100)	Cambridge/UK
**3**	MMP1	ab52631 (Rabbit)/Abcam	1/200	620 (1:100)	Cambridge/UK
**4**	CD3E	ab52959 (Rabbit)/Abcam	1/400	690 (1:100)	Cambridge/UK
**7**	DAPI	C0060/Solarbio	1/500		Beijing/China

Table S2. Procedures for multidimensional fluorescence staining

Antigen	Collagen III	CA9	MMP1	CD3E	DAPI
**Antigen retrieval**	AR6<br>20 min	AR6<br>20 min	AR9<br>20 min	AR6<br>20 min	
**Wash**	TBST<br>10 min	TBST<br>10 min	TBST<br>10 min	TBST<br>10 min	
**Blocking**	10 min<br>@RT	10 min<br>@RT	10 min<br>@RT	10 min<br>@RT	
**Inc. with Primary antibody**	Over night<br>@4℃	Over night<br>@4℃	Over night<br>@4℃	Over night<br>@4℃	10 min<br>@RT
**Wash**	TBST<br>10 min	TBST<br>10 min	TBST<br>10 min	TBST<br>10 min	TBST<br>10 min
**Inc. with Opal HRP Polymer Ms+Rb**	10 min<br>@RT	10 min<br>@RT	10 min<br>@RT	10 min<br>@RT	
**Wash**	TBST<br>10 min	TBST<br>10 min	TBST<br>10 min	TBST<br>10 min	Mounting

@RT: At room temperature; @4℃: At 4℃

Table S3. Results of univariate Cox regression

	HR	P value
**Age**	0.996	0.563
**Gender**		
Female	Reference	
Male	0.972	0.811
**Tumor Location**		**0.001**
Pons	Reference	
Midbrain	0.381	**0.001**
Medulla	0.401	**0.012**
**DIPG**		
N	Reference	
Y	2.882	**<0.001**
**WHO Grade**		
4	Reference	
3	0.344	**0.006**
2	0.076	**<0.001**
1	<0.001	0.971
**Driver Mutation**		**<0.001**
Wildtype	Reference	
H3F3A	10.733	**<0.001**
HIST1H3B	120.301	**<0.001**
IDH1	1.939	0.205
**Radiotherapy**		
Not received	Reference	
Received	0.967	0.892
**Chemotherapy**		
Not received	Reference	
Received	0.624	0.058
